# Inflammasome-independent functions of NAIPs and NLRs in the intestinal epithelium

**DOI:** 10.1042/BST20210365

**Published:** 2021-12-02

**Authors:** Lisa Scarfe, Gillian M. Mackie, Kendle M. Maslowski

**Affiliations:** 1 Institute of Immunology and Immunotherapy, College of Medical and Dental Sciences, University of Birmingham, Birmingham B15 2TT, U.K.; 2 Institute of Metabolism and Systems Research, College of Medical and Dental Sciences, University of Birmingham, Birmingham B15 2TT, U.K.

**Keywords:** epithelial cells, host–pathogen interactions, inflammasome, innate immunity, nod-like receptors

## Abstract

The gut relies on the complex interaction between epithelial, stromal and immune cells to maintain gut health in the face of food particles and pathogens. Innate sensing by the intestinal epithelium is critical for maintaining epithelial barrier function and also orchestrating mucosal immune responses. Numerous innate pattern recognition receptors (PRRs) are involved in such sensing. In recent years, several Nucleotide-binding-domain and Leucine-rich repeat-containing receptors (NLRs) have been found to partake in pathogen or damage sensing while also being implicated in gut pathologies, such as colitis and colorectal cancer (CRC). Here, we discuss the current literature focusing on NLR family apoptosis inhibitory proteins (NAIPs) and other NLRs that have non-inflammasome roles in the gut. The mechanisms behind NLR-mediated protection often converges on similar signalling pathways, such as STAT3, MAPK and NFκB. Further understanding of how these NLRs contribute to the maintenance of gut homeostasis will be important for understanding gut pathologies and developing new therapies.

## Introduction

The intestinal tract is a complex environment, which must achieve both nutrient absorption and protection from pathogens. The combination of food particles, the microbiota, various cell types and potential pathogens requires delicate cross-talk to achieve gut homeostasis and prevent disease. The intestinal epithelium plays a pivotal role in this, acting as a physical barrier and the first line of defence. As such, understanding epithelial-intrinsic immune pathways can provide insight into the mechanisms which limit infections, such as *Salmonella* typhimurium (*S*Tm) and *Shigella*, but also mechanisms that facilitate or exacerbate disease in the context of colorectal cancer (CRC) and inflammatory disorders.

The gut epithelium comprises a single layer of cells, connected via tight junctions. The epithelium produces a mucus layer and secretes anti-microbial peptides to further prevent breach of the barrier. Intestinal epithelial cells (IECs) express a broad arsenal of pattern recognition receptors (PRRs), allowing them to recognise a variety of pathogen and damage associated molecular patterns (PAMPs and DAMPs). The four main families of PRRs include Toll-like receptors (TLRs), retinoic acid-inducible gene 1-like receptors (RLRs), C-type lectin receptors (CLRs) and Nucleotide-binding domain and leucine rich repeat-containing Receptors (NLRs) [1]. A variety of functions occur downstream of receptor activation, many of which signal to cells of the lamina propria beneath the IEC layer, including immune cells.

Here, we focus on the NLR family and in particular NLR family apoptosis inhibitory proteins (NAIPs). Whilst many NLR proteins, including NAIPs, form inflammasomes upon activation, non-inflammasome roles have now been identified for several of the NLR family members. In IECs, a number of NLRs have been linked to disease, in particular CRC. Understanding the diverse epithelial intrinsic roles of NAIPs and other NLR proteins in gut health will enable a more thorough understanding of the signalling which occurs in disease contexts and potentially uncover novel therapeutic targets.

## NLR family apoptosis inhibitory proteins

NAIPs are intracellular PRRs which, as NLR proteins, contain a NACHT domain plus a leucine-rich repeat domain, with three additional N-terminal baculovirus inhibitor of apoptosis (IAP) protein repeat (BIR) domains at the N-terminus [2]. The C57BL/6 genome encodes 4 functional NAIP paralogues which sense components of Gram-negative bacteria. For example, NAIP 1 and 2 can bind the PrgI needle and PrgJ rod subunit of the Type III secretion system (T3SS) of *S*Tm, respectively, whereas NAIP 5 and 6 recognise flagellin [3–5]. Humans express a single NAIP isoform which senses both flagellin and *S*Tm T3SS components [4–7]. Whilst the majority of literature has characterised NAIPs in macrophages, they are also highly expressed in other innate immune cells and IECs [8–10].

### The NAIP/NLRC4 inflammasome

**Figure 1.**
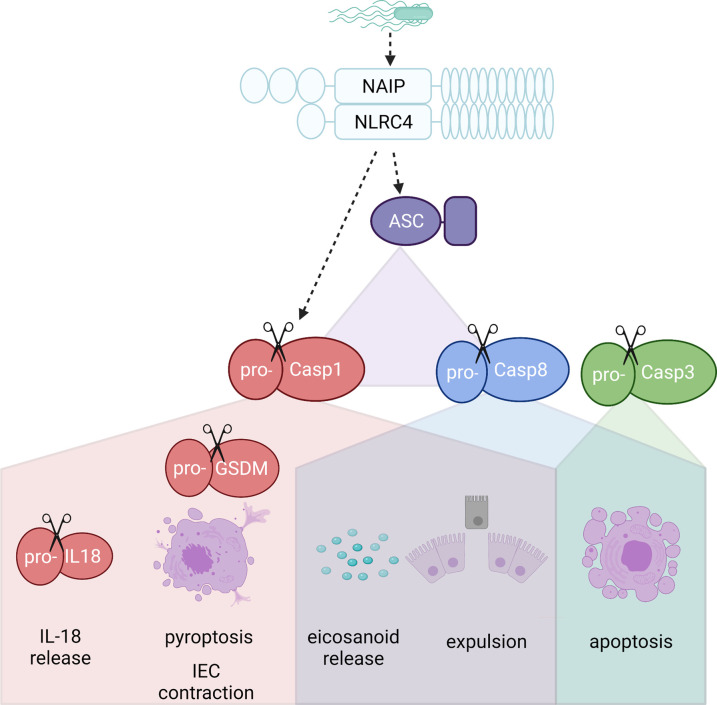
The intestinal epithelial cell-specific effects the NAIP/NLRC4 inflammasome. Upon activation by components of gram negative bacteria, NAIP co-oligomerises with NLRC4 to form the inflammasome. NLRC4 can either bind pro-caspase-1 directly, or bind apoptosis speck-like protein (ASC), an adaptor protein, which subsequently recruits pro-caspase-1 or pro-caspase-8, resulting in the proteolytic cleavage and activation of the pro-caspases. Redundancy exists between the caspases; whilst both caspase-1 and caspase-8 mediate IEC death and expulsion, caspase-1 triggers a more inflammatory pyroptosis-like death whereas caspase-8 mediates apoptosis-like cell death [2]. Caspase-3, an executor caspase which is activated by other caspases, has also been found to be activated following NAIP activation, which would lead to apoptotic cell death [21]. Either caspase-1 or -8 has also been shown to be sufficient to trigger eicosanoid release following NAIP activation [12]. Unique to caspase-1 activity is cleavage and activation of pro-Gasdermin-D which triggers pyroptosis and IEC contraction via the formation of pores in the plasma membrane [23,27]. Caspase-1 also proteolytically cleaves and activates IL-18. In other cell types, such as macrophages, IL-1β is also released [6]. Figure created in Biorender.com.

Upon ligand binding, NAIPs oligomerize with NLR family CARD-containing 4 (NLRC4) to form the NAIP/NLRC4 inflammasome. This is one of several canonical inflammasomes, which usually consist of caspase-1, the adaptor protein ASC (apoptosis speck-like protein), plus an NLR protein (e.g. NLRP1, NLRP3, NLRC4, NLRP6 or NLRP12) or proteins such as Absent in Melanoma 2 (AIM2) [11]. NLRC4 then directly recruits pro-caspase-1 via the association of the CARD domains, or alternatively via the adaptor protein ASC, which allows it to recruit pro-caspase-1 or pro-caspase-8 (Figure 1) [2,12]. The pro-caspases are then cleaved and activated [11]. In macrophages, Caspase-1 proteolytically cleaves and activates pro-IL-1β, pro-IL-18 and Gasdermin D - the latter forming pores in the plasma membrane of the cell, resulting in pyroptosis and release of IL-1β and IL-18 [13–17]. Caspase-8 activation leads to apoptotic cell death, with IL-1β, IL-18 and other alarmins retained within the cell, resulting in a less inflammatory form of cell death [18]. This caspase-8 mediated apoptosis can be inhibited by TLR signalling, via expression of c-FLIP - a process leaving NLRC4-mediated pyroptosis unaffected [18]. In addition, Caspase-3 has recently been reported to be activated by the NAIP/NLRC4 inflammasome. Caspase-3, an executioner caspase, is canonically activated by initiator caspases, such as Caspase-8, resulting in apoptotic cell death (18).

In IECs, pathogen detection by NAIPs leads to expulsion of the infected cell into the gut lumen and cell death [9] (Figure 1). Whilst this causes moderate enteropathy, it acts as a critical first line of defence in limiting bacterial dissemination. This was illustrated by Hausmann et al. using wild-type (WT) isogenic tagged strains of *S*Tm to determine dissemination to the mesenteric lymph nodes. Increased numbers of *S*Tm were found in *Nlrc4^−^*^/*−*^ and IEC-specific NAIP knock-out mice (*Naip1–6^Δ^*^/ΔIEC^), however this effect was lost when using a *S*Tm strain which bypasses the IECs. The NAIP ligands, flagellin and the T3SS, are required for IEC invasion and are subsequently down-regulated to avoid immune detection. Thus, NAIPs expressed in the IECs, as opposed to the innate immune cells, are vital in the initial detection of *Salmonella* infection [19]. A separate study showed that lack of Caspase-1 caused increased *S*Tm burden and reduced epithelial cell expulsion, with Caspase-11 only having an effect under inflammatory conditions, generated by pre-treatment with IFNγ, further highlighting the importance of this pathway in early infection [20]. In the absence of this mechanism, *S*Tm-infected mice have excessive IEC loss and collapse of the epithelial barrier at later time points, due to TNF release by bone marrow-derived cells [21].

NAIP-mediated cell death appears to take on both apoptotic and pyroptotic qualities in IECs. Mice treated with potent NAIP activator FlaTox exhibited fluid loss, vascular leakage and diarrhoea. Initial experiments using FlaTox, a potent NAIP5–6 activator which delivers flagellin to the cytosol of cells, found that lysis of IECs occurred prior to cell expulsion in a manner similar to pyroptosis [12]. This effect was lost if both caspase-1 and caspase-8, or caspase-1 alone, was ablated [12]. However, *S*Tm infection of intestinal organoids has since found that plasma membrane integrity is lost at varying stages, most often after expulsion, as well as caspase-3 cleavage [21]. Taken together, these results suggest a combination of inflammatory and apoptotic cell death occurs, potentially with activation of multiple caspases simultaneously and redundancy between the caspases [12,18,21]. This heterogenous activation of cell death pathways is in line with the emerging concept of ‘PAN-optosis’ [22]. The resultant gap created by cell expulsion is closed by cells in an actomyosin-dependent manner, allowing the epithelial barrier to be maintained. If actin polymerisation is blocked no expulsion occurs [12,23]. In addition, contractions of IECs occur which densely packs cells at the site of infection. Sub-lytic levels of Gasdermin D-mediated pore formation are thought to lead to ion fluxes which trigger these non-muscle myosin II contractions. This contraction was shown in both mouse and human IEC monolayers, and was lost if NAIPs, NLRC4, caspase-1 or Gasdermin D were ablated as well as in the presence of blebbistatin or Gd3+, which blocks myosin and ion fluxes caused by Gasdermin D pores, respectively [23].

Alongside cell death and expulsion, NAIP/NLRC4 activation in IECs results in release of IL-18 and eicosanoids [12,24]. Mice treated with potent NAIP activator FlaTox suffered fluid loss, vascular leakage and diarrhoea, with *Nlrc4^−^*^/*−*^, *Naip5^−^*^/*−*^ and *Caspase-1^−^*^/*−*^ mice being protected from this effect [24]. Eicosanoid release had been suggested to be a result of caspase-1-mediated Ca^2+^ influx into peritoneal macrophages, resulting in activation of cPLA_2_, the enzyme upstream of eicosanoid biosynthesis [24]. However, intestinal tissue PGE_2_ release following FlaTox treatment has since been shown to be comparable between WT mice and mice with NLRC4 expression restricted to the IECs, suggesting that eicosanoids release can be mediated by the IECs [12]. The mechanism of NAIPs/NLRC4-mediated eicosanoid release hasn't been explored, but could feasibly be similar to that described in macrophages (caspase-1-mediated Ca^2+^ influx).

### NAIPs as tumour suppressors

Aside from their roles involving the NLRC4 inflammasome, NAIPs have also been shown to function as tumour suppressors in CRC. Allam et al. found that mice with all NAIP isoforms deleted (*Naip1–6*^Δ/Δ^) were protected from dextran sulfate sodium (DSS)-induced colitis, but had greater tumour burden following treatment with the carcinogen azoxymethane (AOM) alone, and in the AOM/DSS model of colitis-associated cancer. The effect was epithelial NAIP-mediated, as IEC-specific KO had increased AOM/DSS-induced tumourigenesis whereas knockout in the myeloid compartment had comparable tumour burden as littermate controls [8]. During DSS-induced colitis, *Naip1–6*^Δ/Δ^ mice exhibited reduced pro-inflammatory cytokine transcripts (*Il-1α/β*, *-6*, *-17*, *Cxcl-1*) and increased anti-apoptotic and survival transcripts (*Bcl-2*, *Myc*, *Mdm2*, *Ccnd1* and *Il-22*), suggesting increased repair following damage and limitation of inflammation [8]. Interestingly, *Naip1–6*^Δ/Δ^ mice failed to activate p53 and apoptosis in the early response to carcinogen. *Naip1–6*^Δ/Δ^ mice also had increased STAT3 phosphorylation following AOM exposure compared with *Naip1–6*^fl/fl^ controls; which was absent from *Nlrc4^−^*^/*−*^, *Caspase-1/11^−^*^/*−*^ or *Asc^−^*^/*−*^ mice, implying no involvement of inflammasome signalling in this phenotype [8]. Additionally, production of IL-1β or IL-18 was not affected in *Naip1–6^Δ/Δ^* mice after AOM nor during active colitis or in tumours, further implying that inflammasome activation is not involved the phenotype of *Naip1–6*^Δ/Δ^ mice (8). Indeed, it would not be expected that known activators of the NAIP/NLRC4 inflammasome (i.e. pathogen components) would be present in the context of carcinogen or DSS, though it is feasible that a component of the microbiota could provide such a signal [8]. Other studies have reported that *Caspase1^−^*^/*−*^ and *Nlrc4^−^*^/*−*^ mice also have increased tumour burden following AOM/DSS treatment, but exhibited similar colitis symptoms to WT, in contrast with *Naip1–6*^Δ/Δ^ mice [8,25], suggesting that NAIPs, NLRC4 and caspases might not only have differing roles during tumour induction, but also likely involve multiple different pathways. It is worth noting that these studies often did not use littermate controls, which could influence the susceptibility to colitis and therefore CRC. In the absence of side-by-side knock-out experiments of the different inflammasome components (i.e. NAIPs, NLRC4, ASC, Caspase-1) using corresponding littermate controls, it is difficult to unpick whether the effect of NAIPs in cancer is driven exclusively by non-inflammasome or inflammasome mechanisms, or a combination of the two. In addition, due to the difficulty of generating quadruple KO mice, Allam et al. deleted the entire *Naip* locus. Thus the possibility of off-target effects due to deletion of unknown regulatory elements in the non-coding regions cannot be excluded. The role of individual NAIPs could also be investigated in the future as it is unclear how much redundancy exists between the different paralogues in the context of colitis and CRC. NAIPs have also been shown to be down-regulated in mouse and human colorectal tumours, which could imply loss of cell types expressing NAIPs or an active repression of what might be considered an innate tumour suppressor to aid tumour escape [8,26].

### Other NLR family members in CRC

**Figure 2.**
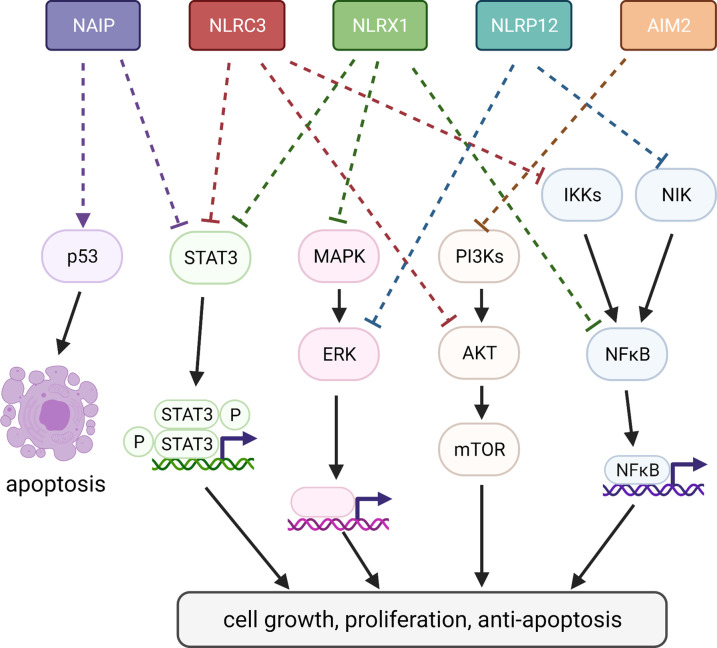
Inflammasome-independent signalling by NLR proteins in the intestinal epithelium during CRC. The NLR proteins NAIP, NLRC3, NLRX1, NLRP12 and AIM2 have all been shown to protect against CRC via inflammasome-independent pathways in the intestinal epithelium. Following NAIP knockout, epithelial cells fail to activate p53 and have elevated STAT3 phosphorylation following AOM exposure [8]. *Nlrc3*^−/−^ mice had elevated STAT3 phosphorylation, IκBα and AKT levels following AOM/DSS exposure [51]. *Apc^Min/+^Nlrx1*^−/−^ mice had increased activation of NFκB, MAPK, STAT3 [33]. NLRP12 deletion resulted in non-canonical NFκB activation via NFκB-induced kinase (NIK) and increased ERK signalling, which affects multiple TFs [40,41]. AIM2 has been shown to suppress the PI3K/AKT pathway [44,45,47]. These pathways promote cancer via transcription of cell growth, proliferation and anti-apoptosis genes. Figure created in Biorender.com.

A number of NLR proteins have non-inflammasome functions, including NLRP10, NLRC3, NLRC5, NLRX1 and CIITA. These NLRs often regulate transcription of the NFκB, MAPK and major histocompatibility complex (MHC) signalling pathways [27,28]. A number of NLR family protein have been linked to cancer development (Figure 2). Increased tumorigenesis following AOM/DSS exposure is seen in *Nlrc3^−^*^/*−*^, *Nlrp3^−^*^/*−*^, *Nlrp6^−^*^/*−*^, *Nlrc4^−^*^/*−*^, *Nlrp1^−^*^/*−*^, *Nlrx1^−^*^/*−*^ and *Nlrp12^−^*^/*−*^ mice [27]. Many NLR proteins have been linked to cancer development via inflammasome-dependent pathways [29], including NLRC4 [25] and NLRP1 [30]. *Caspase-1^−^*^/*−*^ and *Asc^−^*^/*−*^ mice have also been shown to have increased tumorigenesis following AOM/DSS exposure [25,29]. Loss of IL-18 release is thought to at least partially mediate this, with *Il18^−^*^/*−*^ and *Il18r1^−^*^/*−*^ mice also having increased tumorigenesis following AOM/DSS exposure, along with decreased expression of DNA damage repair genes *Atm*, *Atr*, *Msh1* and *Parp1* [29]. In *Caspase-1^−^*^/*−*^ mice, administration of IL-18 reduced signs of inflammation, ulceration and hyperplasia in AOM/DSS treated mice [29,31]. As NLR proteins are expressed in multiple cell types in the colon, both haematopoietic and non-haematopoietic compartments have been implicated in their tumour suppressor functions. Here onwards, we focus on non-inflammasome roles of NLR proteins in the IECs (Figure 2).

#### NLRX1

NLRX1 is unusual in the NLR family in that it does not form an inflammasome; conversely it is thought to negatively regulate inflammatory responses [32]. Studies have found NLRX1 in IECs to have a tumour suppressor role, and human colon tumours also have reduced NLRX1 expression [33,34]. Tumorigenesis is increased in both whole-body and IEC-specific *Nlrx1* deletion in the AOM/DSS model and in *Apc^min/+^* mice, which sporadically develop intestinal polyps [32–34]. IEC-specific knock out of *Nlrx1* resulted in increased *Tnf*, *Egf* and *Tgfb1* expression, all of which are associated with healing and proliferation in the intestinal epithelium following injury [35,36]. Accordingly, these mice recovered faster following DSS treatment, despite comparable inflammation to WT littermates [33]. Tattoli et al., concluded that this phenotype was due to increased TNF signalling, with TNF-stimulated intestinal organoids exhibiting increased proliferation and expression of stem cell markers *Olfm4* and *Myb* [33]. Another study found that compared with *Apc^min/+^* littermates, *Apc^min/+^Nlrx1^−^*^/*−*^ mice had increased activation of NFκB, MAPK, STAT3, β-catenin, and cathepsin B*,* plus increased IL-6 levels. Treatment with anti-IL-6R antibody reduced mortality and tumorigenesis and decreased STAT3 activation in *Apc^Min/+^Nlrx1^−^*^/*−*^ mice. Koblansky et al. therefore proposed that NLRX1 inhibited MAPK and NFκB signalling, which would otherwise lead to IL-6 release and subsequent STAT3 phosphorylation [34]. The discrepancies between these two studies may be explained by the fact that one used whole-body *Nlrx1* knock out and the other specifically ablated *Nlrx1* in the IECs, meaning myeloid cell-derived NLRX1 could still have an effect. Nevertheless, these reports indicate that NLRX1 has roles in suppressing intestinal tumour formation with functions directly attributed to IEC-intrinsic NLRX1 expression [33,34].

#### NLRP12

NLRP12 is generally thought to act as a negative regulator of NFκB signalling, but also detects *Yersinia* pestis infection to induce inflammasome signalling [37–39]. *Nlrp12^−^*^/*−*^ mice are more susceptible to colitis and colitis-associated cancer, due to heightened NFκB and ERK signalling [40,41]. Bone marrow-derived dendritic cells isolated from *Nlrp12^−^*^/*−*^ mice had sustained NFκB-induced kinase (NIK) activation and decreased levels of TRAF3, which normally functions to regulate non-canonical NFκB signalling. Different studies have reached opposing conclusions as to whether the haematopoietic or non-haematopoietic compartment are responsible for the phenotype of *Nlrp12^−^*^/*−*^ mice, highlighting that NLRs in numerous cell types can play a role in cancer development [40,41]. Elevated expression of various cancer-associated genes, such as *Akt1*, *Jun*, *Nr3c1* and the NIK-regulated genes, *Cxcl12* and *Cxcl13,* has also been observed in *Nlrp12^−^*^/*−*^ colons following AOM/DSS exposure [40]. However, it is worth noting that neither of these studies used littermate controls. Differences in the microbiota strongly influence the development of inflammation following DSS treatment [42]. DSS-induced inflammation is a major driver of carcinogenesis in the AOM/DSS model, meaning further studies would be required to determine if microbiota differences were affecting these results [43]. Previous studies have already determined that *Nlrp12^−^*^/*−*^ mice have an altered microbiome due to increased basal colonic inflammation, leading to increased colitis susceptibility, further highlighting the importance of the microbiome in the development of inflammatory diseases [44].

#### AIM2

AIM2 recognises cytosolic DNA and forms an inflammasome, resulting in caspase-1 activation [45]. A reduction in AIM2 expression has been observed in a number of human cancers, including CRC [46]. Loss of AIM2 has also been shown to increase tumorigenesis in both the AOM/DSS and *Apc^Min/−^* models of CRC [45,47]. This effect appeared to be independent of the inflammasome as levels of IL-1β and IL-18 are comparable to WT [45,47]. Several studies have found AIM2 to suppress the PI3K/Akt pathway [45,47,48], and Wilson et al. [47] showed that AIM2 associated with and reduced the activation of DNA-PK, a kinase which phosphorylates Akt. This appeared to be mediated by epithelial cells as *Aim2^−^*^/*−*^ epithelial organoids stimulated by Insulin-like Growth Factor-1 (IGF-1), known to activate the Akt signalling pathway, had elevated p-Akt compared with WT. In contrast, no difference was seen in p-Akt levels in IGF-1- or lipopolysaccharide-stimulated WT versus *Aim2^−^*^/*−*^ bone-marrow derived macrophages [47]. The absence of PI3K/Akt pathway suppression by AIM2 has been shown to decrease apoptosis in HCT116 CRC cells [48], as well as increase the capacity of stem cells to form organoids in culture [45]. However, other studies have suggested that AIM2 may regulate CRC via caspase-1-mediated induction of cell death and regulation of the epithelial-to-mesenchymal transition [49,50].

#### NLRC3

Two independent studies have shown NLRC3 has tumour suppressive roles in IECs [51,52]. *Nlrc3^−^*^/*−*^ mice are more susceptible to DSS-induced colitis and had greater tumorigenesis in both the AOM/DSS and *Apc^Min/+^* model. *Nlrc3^−^*^/*−*^ mice cohoused with WT were as susceptible to tumorigenesis as separately housed *Nlrc3^−^*^/*−*^ mice, indicating that susceptibility to tumorigenesis was not due to microbiota changes [52]. Specific knock out of *Nlrc3* in the IECs also resulted in increased tumorigenesis following AOM/DSS compared with knock out in the myeloid or haematopoietic compartment. Following AOM/DSS treatment, *Nlrc3^−^*^/*−*^ mice had elevated levels of IκBα and STAT3 phosphorylation and increased macrophage, neutrophil and NK cell influx in the colon. Elevated Ki67 and PCNA, indicating cell proliferation, and increased phosphorylation of S6 kinase and AKT, downstream targets of mTOR, were observed in the IECs [51]. Stem cells from both AOM/DSS-treated *Nlrc3^−^*^/*−*^ and *Apc^Min/+^Nlrc3^−^*^/*−*^ more readily formed organoids compared with WT and *Apc^Min/+^* mice, respectively; indicative of being more prone to tumorigenesis [51]. Stem cell markers, such as OLFM4 and SOX9, were also increased in colons of *Nlrc3^−^*^/*−*^ mice compared with WT following AOM exposure [52]. Treatment of *Apc^Min/+^Nlrc3^−^*^/*−*^ mice with a PI3K/mTOR inhibitor reduced organoid-forming capacity, tumour burden and phosphorylation of S6 kinase, indicating a mechanism by which NLRC3 maintains homeostasis and limits tumourigenesis. Furthermore, NLRC3 was found to co-immunoprecipitate with subunit p85 of PI3K, leading to the hypothesis that NLRC3 disrupts the association between subunits p85 and p110a, thus reducing PI3K activity and suppressing tumorigenesis [51].

### Future considerations

The cellular roles of NLRs have been studied in great detail in innate phagocytes, and are increasingly being studied in intestinal epithelial cells. The role of NAIPs in gram-negative bacteria infection have been well established, however the mechanisms by which NAIPs influence CRC and colitis remain ill-defined. An outstanding question is how NLR functions, inflammasome-dependent and independent, impact on the underlying mucosal-associated lymphoid tissue (MALT). The link between altered inflammatory mediators and changes in mucosal immune responses in the context of IEC-specific deletion of NLR proteins has been little studied. As discussed, *Nlrx1^−^*^/*−*^ mice had increased TNF or IL-6 production [33,34], which could invariably affect immune infiltration and function. Bacterial-mediated NAIP/NLRC4 activation in IECs led to robust IL-18 and eicosanoid production, particularly PGE_2_, which can promote Th1 and Th17 cell differentiation and function [12,53]. It remains to be determined whether eicosanoids might be affected during AOM and DSS insults in a NAIPs-dependent manner. Clearly there are multiple avenues by which NLRs could be influencing the immune compartment during CRC and colitis, but further work is required to establish these mechanisms. Additionally, little is known whether there is a homeostatic role for these NLRs, particularly those with inflammasome-independent function as these could conceivably be active in the steady state. Crucially, any future work investigating the role of IEC-expressed NLRs in carcinogenesis will need to prioritise the use of littermate controls, due to the influence the microbiota can have on the development of inflammation in models such as AOM/DSS treatment [42]. A study of *Nlrp6^−^*^/*−*^ mice using littermate controls highlighted this necessity, finding that *Nlrp6* knock out had no effect on the microbiome or susceptibility to DSS-induced colitis, in contrast with previous studies which did not use littermate controls [54].

### Conclusion

The gut is a unique environment, relying on the integrated communication between various cell types. As such, the downstream consequences of PRR activation can have a wide impact on various signalling pathways, in some cases with distinct effects in specific cell types [1]. The epithelial-intrinsic effects of NAIPs are pivotal to the immune response against pathogens such as *S*Tm; epithelial-expressed NAIPs are in a unique position to exploit the bacterial requirement for flagellin and the T3SS to reach and infect epithelial cells, activating NLRC4 and downstream inflammasome signalling [9,12,19,21]. But similar to several other NLR proteins, NAIPs also suppress tumour formation in the colon, which appears to not require NLRC4 inflammasome signalling [8]. NAIPs, NLRC3, NLRX1, NLRP12 and AIM2 all suppress tumourigenesis in an epithelial-intrinsic manner. There are recurring observations across studies, including activation of STAT3, PI3K, mTOR, Akt and NFκB (illustrated in Figure 2) [32–34,40,47,51,55]. Together, these studies highlight the importance of epithelial NLR proteins in CRC development. Understanding these pathways in greater detail will help clarify the complex sequence of events that lead to and exacerbate CRC, thus enabling the development of more effective therapies.

## Perspectives

- NAIPs have epithelial-intrinsic functions to control *Salmonella* infection, with inflammasome activation inducing a range of responses not typical to that seen in macrophages.
- NAIPs and other NLRs have non-inflammasome functions in the intestinal epithelium which protect from tumourigenesis, suggestive of innate immune checkpoints to cancer development.
- Further understanding of epithelial NLR functions in maintaining homeostasis and effects on mucosal immune status is warranted.

## Open Access Statement

Open access for this article was enabled by the participation of University of Birmingham in an all-inclusive Read & Publish pilot with Portland Press and the Biochemical Society under a transformative agreement with JISC.

## Abbreviations

AIM2: Absent in melanoma 2
AOM: Axozymethane
CARD: Caspase activation and recruitment domain
Casp: Caspase
CRC: Colorectal cancer
DSS: Dextran sulfate sodium
GSDM: Gasdermin D
IECs: Intestinal epithelial cells
NAIPs: NLR family apoptosis inhibitory proteins (formerly known as ‘Neuronal apoptosis inhibitor proteins’) [56].
NLR: Nucleotide binding domain and leucine rich repeat-containing receptors
NLRC4: Nod-Like Receptor family, CARD-containing 4
PAMPs/DAMPS: Pattern/Damage-associated molecular patterns
PRRs: Pattern recognition receptors
STm: *Salmonella* typhimurium
T3SS: Type III secretion system
TLRs: Toll-like receptors
WT: Wild-type

## References

[1] Amarante-Mendes, G.P., Adjemian, S., Branco, L.M., Zanetti, L.C., Weinlich, R. and Bortoluci, K.R. (2018) Pattern recognition receptors and the host cell death molecular machinery. Front. Immunol. 9, 2379 10.3389/fimmu.2018.02379 30459758 PMC6232773

[2] Bauer, R. and Rauch, I. (2020) The NAIP/NLRC4 inflammasome in infection and pathology. Mol. Aspects Med. 76, 100863 10.1016/j.mam.2020.100863 32499055

[3] Kofoed, E.M. and Vance, R.E. (2011) Innate immune recognition of bacterial ligands by NAIPs determines inflammasome specificity. Nature 477, 592–595 10.1038/nature10394 21874021 PMC3184209

[4] Zhao, Y., Yang, J., Shi, J., Gong, Y.-N., Lu, Q., Xu, H. et al. (2011) The NLRC4 inflammasome receptors for bacterial flagellin and type III secretion apparatus. Nature 477, 596–600 10.1038/nature10510 21918512

[5] Yang, J., Zhao, Y., Shi, J. and Shao, F. (2013) Human NAIP and mouse NAIP1 recognize bacterial type III secretion needle protein for inflammasome activation. Proc. Natl Acad. Sci. U.S.A. 110, 14408–14413 10.1073/pnas.1306376110 23940371 PMC3761597

[6] Kortmann, J., Brubaker, S.W. and Monack, D.M. (2015) Cutting edge: inflammasome activation in primary human macrophages is dependent on flagellin. J. Immunol. 195, 815–819 10.4049/jimmunol.1403100 26109648 PMC4505955

[7] Ruiz, V.M.R., Ramirez, J., Naseer, N., Palacio, N.M., Siddarthan, I.J., Yan, B.M. et al. (2017) Broad detection of bacterial type III secretion system and flagellin proteins by the human NAIP/NLRC4 inflammasome. Proc. Natl Acad. Sci. U.S.A. 114, 13242–13247 10.1073/pnas.1710433114 29180436 PMC5740664

[8] Allam, R., Maillard, M.H., Tardivel, A., Chennupati, V., Bega, H., Yu, C.W. et al. (2015) Epithelial NAIPs protect against colonic tumorigenesis. J. Exp. Med. 212, 369–383 10.1084/jem.20140474 25732303 PMC4354369

[9] Sellin, M.E., Müller, A.A., Felmy, B., Dolowschiak, T., Diard, M., Tardivel, A. et al. (2014) Epithelium-intrinsic NAIP/NLRC4 inflammasome drives infected enterocyte expulsion to restrict Salmonella replication in the intestinal mucosa. Cell Host Microbe. 16, 237–248 10.1016/j.chom.2014.07.001 25121751

[10] Diez, E., Yaraghi, Z., MacKenzie, A. and Gros, P. (2000) The neuronal apoptosis inhibitory protein (Naip) is expressed in macrophages and is modulated after phagocytosis and during intracellular infection with legionella pneumophila. J. Immunol. 164, 1470–1477 10.4049/jimmunol.164.3.1470 10640764

[11] Winsor, N., Krustev, C., Bruce, J., Philpott, D.J. and Girardin, S.E. (2019) Canonical and noncanonical inflammasomes in intestinal epithelial cells. Cell Microbiol. 21, e13079 10.1111/cmi.13079 31265745

[12] Rauch, I., Deets, K.A., Ji, D.X., von Moltke, J., Tenthorey, J.L., Lee, A.Y. et al. (2017) NAIP-NLRC4 Inflammasomes coordinate intestinal epithelial cell expulsion with eicosanoid and IL-18 release via activation of caspase-1 and -8. Immunity 46, 649–659 10.1016/j.immuni.2017.03.016 28410991 PMC5476318

[13] Shi, J., Zhao, Y., Wang, K., Shi, X., Wang, Y., Huang, H. et al. (2015) Cleavage of GSDMD by inflammatory caspases determines pyroptotic cell death. Nature 526, 660–665 10.1038/nature15514 26375003

[14] Franchi, L., Amer, A., Body-Malapel, M., Kanneganti, T.-D., Özören, N., Jagirdar, R. et al. (2006) Cytosolic flagellin requires Ipaf for activation of caspase-1 and interleukin 1β in salmonella-infected macrophages. Nat. Immunol. 7, 576–582 10.1038/ni1346 16648852

[15] Miao, E.A., Alpuche-Aranda, C.M., Dors, M., Clark, A.E., Bader, M.W., Miller, S.I. et al. (2006) Cytoplasmic flagellin activates caspase-1 and secretion of interleukin 1β via Ipaf. Nat. Immunol. 7, 569–575 10.1038/ni1344 16648853

[16] Molofsky, A.B., Byrne, B.G., Whitfield, N.N., Madigan, C.A., Fuse, E.T., Tateda, K. et al. (2006) Cytosolic recognition of flagellin by mouse macrophages restricts Legionella pneumophila infection. J. Exp. Med. 203, 1093–1104 10.1084/jem.20051659 16606669 PMC1584282

[17] Miao, E.A., Mao, D.P., Yudkovsky, N., Bonneau, R., Lorang, C.G., Warren, S.E. et al. (2010) Innate immune detection of the type III secretion apparatus through the NLRC4 inflammasome. Proc. Natl Acad. Sci. U.S.A. 107, 3076–3080 10.1073/pnas.0913087107 20133635 PMC2840275

[18] Van Opdenbosch, N., Van Gorp, H., Verdonckt, M., Saavedra, P.H.V., de Vasconcelos, N.M., Gonçalves, A. et al. (2017) Caspase-1 engagement and TLR-induced c-FLIP expression suppress ASC/Caspase-8-dependent apoptosis by inflammasome sensors NLRP1b and NLRC4. Cell Rep. 21, 3427–3444 10.1016/j.celrep.2017.11.088 29262324 PMC5746600

[19] Hausmann, A., Böck, D., Geiser, P., Berthold, D.L., Fattinger, S.A., Furter, M. et al. (2020) Intestinal epithelial NAIP/NLRC4 restricts systemic dissemination of the adapted pathogen Salmonella Typhimurium due to site-specific bacterial PAMP expression. Mucosal Immunol. 13, 530–544 10.1038/s41385-019-0247-0 31953493 PMC7181392

[20] Crowley, S.M., Han, X., Allaire, J.M., Stahl, M., Rauch, I., Knodler, L.A. et al. (2020) Intestinal restriction of Salmonella Typhimurium requires caspase-1 and caspase-11 epithelial intrinsic inflammasomes. PLoS Pathog. 16, e1008498 10.1371/journal.ppat.1008498 32282854 PMC7179941

[21] Fattinger, S.A., Geiser, P., Samperio Ventayol, P., Di Martino, M.L., Furter, M., Felmy, B. et al. (2021) Epithelium-autonomous NAIP/NLRC4 prevents TNF-driven inflammatory destruction of the gut epithelial barrier in Salmonella -infected mice. Mucosal Immunol. 14, 615–629 10.1038/s41385-021-00381-y 33731826 PMC8075861

[22] Malireddi, R.K.S., Kesavardhana, S. and Kanneganti, T.-D. (2019) ZBP1 and TAK1: master regulators of NLRP3 inflammasome/pyroptosis, apoptosis, and necroptosis (PAN-optosis). Front. Cell. Infect. Microbiol. 9, 406 10.3389/fcimb.2019.00406 31850239 PMC6902032

[23] Ventayol, P.S., Geiser, P., Martino, M.L.D., Florbrant, A., Fattinger, S.A., Walder, N. et al. (2021) Bacterial detection by NAIP/NLRC4 elicits prompt contractions of intestinal epithelial cell layers. Proc. Natl Acad. Sci. U.S.A. 118, e2013963118 10.1073/pnas.2013963118 33846244 PMC8072224

[24] von Moltke, J., Trinidad, N.J., Moayeri, M., Kintzer, A.F., Wang, S.B., van Rooijen, N. et al. (2012) Rapid induction of inflammatory lipid mediators by the inflammasome in vivo. Nature 490, 107–111 10.1038/nature11351 22902502 PMC3465483

[25] Hu, B., Elinav, E., Huber, S., Booth, C.J., Strowig, T., Jin, C. et al. (2010) Inflammation-induced tumorigenesis in the colon is regulated by caspase-1 and NLRC4. Proc. Natl Acad. Sci. U.S.A. 107, 21635–21640 10.1073/pnas.1016814108 21118981 PMC3003083

[26] Endo, T., Abe, S., Seidlar, H.B.K., Nagaoka, S., Takemura, T., Utsuyama, M. et al. (2004) Expression of IAP family proteins in colon cancers from patients with different age groups. Cancer Immunol. Immunother. 53, 770–776 10.1007/s00262-004-0534-8 15138717 PMC11034232

[27] Zhu, H. and Cao, X. (2017) NLR members in inflammation-associated carcinogenesis. Cell. Mol. Immunol. 14, 403–405 10.1038/cmi.2017.14 28366939 PMC5423091

[28] Meissner, T.B., Li, A., Biswas, A., Lee, K.-H., Liu, Y.-J., Bayir, E. et al. (2010) NLR family member NLRC5 is a transcriptional regulator of MHC class I genes. Proc. Natl Acad. Sci. U.S.A. 107, 13794–13799 10.1073/pnas.1008684107 20639463 PMC2922274

[29] Pandey, A., Shen, C. and Man, S.M. (2019) Inflammasomes in colitis and colorectal cancer: mechanism of action and therapies. Yale J. Biol. Med. 92, 481–498 PMID:31543710 PMC6747943

[30] Williams, T.M., Leeth, R.A., Rothschild, D.E., Coutermarsh-Ott, S.L., McDaniel, D.K., Simmons, A.E. et al. (2015) The NLRP1 inflammasome attenuates colitis and colitis-associated tumorigenesis. J. Immunol. 1950 194, 3369–3380 10.4049/jimmunol.1402098 PMC4369420 25725098

[31] MdH, Z., Vogel, P., Body-Malapel, M., Lamkanfi, M. and Kanneganti, T.-D. (2010) IL-18 production downstream of the Nlrp3 inflammasome confers protection against colorectal tumor formation. J. Immunol. 1950 185, 4912–4920 10.4049/jimmunol.1002046 PMC3104023 20855874

[32] Lei, A. and Maloy, K.J. (2016) Colon cancer in the land of NOD: NLRX1 as an intrinsic tumor suppressor. Trends Immunol. 37, 569–570 10.1016/j.it.2016.07.004 27452288

[33] Tattoli, I., Killackey, S.A., Foerster, E.G. Molinaro, R., Maisonneuve, C., Rahman, M.A. et al. (2016) NLRX1 acts as an epithelial-Intrinsic tumor suppressor through the modulation of TNF-Mediated proliferation. Cell Rep. 14, 2576–2586 10.1016/j.celrep.2016.02.065 26971996

[34] Koblansky, A.A., Truax, A.D., Liu, R., Montgomery, S.A., Ding, S., Wilson, J.E. et al. (2016) The innate immune receptor NLRX1 functions as a tumor suppressor by reducing colon tumorigenesis and key tumor-promoting signals. Cell Rep. 14, 2562–2575 10.1016/j.celrep.2016.02.064 26971998 PMC4853907

[35] Sturm, A. and Dignass, A.U. (2008) Epithelial restitution and wound healing in inflammatory bowel disease. World J. Gastroenterol. 14, 348–353 10.3748/wjg.14.348 18200658 PMC2679124

[36] Leppkes, M., Roulis, M., Neurath, M.F., Kollias, G. and Becker, C. (2014) Pleiotropic functions of TNF-α in the regulation of the intestinal epithelial response to inflammation. Int. Immunol. 26, 509–515 10.1093/intimm/dxu051 24821262

[37] Vladimer, G.I., Weng, D., Paquette, S.W.M., Vanaja, S.K., Rathinam, V.A.K., Aune, M.H. et al. (2012) The NLRP12 inflammasome recognizes yersinia pestis. Immunity 37, 96–107 10.1016/j.immuni.2012.07.006 22840842 PMC3753114

[38] Williams, K.L., Lich, J.D., Duncan, J.A., Reed, W., Rallabhandi, P., Moore, C. et al. (2005) The CATERPILLER protein monarch-1 is an antagonist of toll-like receptor-, tumor necrosis factor alpha-, and *Mycobacterium tuberculosis*-induced pro-inflammatory signals. J. Biol. Chem. 280, 39914–39924 10.1074/jbc.M502820200 16203735 PMC4422647

[39] Lich, J.D., Williams, K.L., Moore, C.B., Arthur, J.C., Davis, B.K., Taxman, D.J. et al. (2007) Monarch-1 suppresses non-canonical NF-kappaB activation and p52-dependent chemokine expression in monocytes. J. Immunol. 1950 178, 1256–1260 10.4049/jimmunol.178.3.1256 17237370

[40] Allen, I.C., Wilson, J.E., Schneider, M., Lich, J.D., Roberts, R.A., Arthur, J.C. et al. (2012) NLRP12 suppresses colon inflammation and tumorigenesis through the negative regulation of noncanonical NF-κB signaling. Immunity 36, 742–754 10.1016/j.immuni.2012.03.012 22503542 PMC3658309

[41] MdH, Z., Vogel, P., Malireddi, R.K.S., Body-Malapel, M., Anand, P.K., Bertin, J. et al. (2011) The NOD-Like receptor NLRP12 attenuates colon inflammation and tumorigenesis. Cancer Cell 20, 649–660 10.1016/j.ccr.2011.10.022 22094258 PMC3761879

[42] Rosshart, S.P., Vassallo, B.G., Angeletti, D., Hutchinson, D.S., Morgan, A.P., Takeda, K. et al. (2017) Wild mouse Gut microbiota promotes host fitness and improves disease resistance. Cell 171, 1015–1028.e13 10.1016/j.cell.2017.09.016 29056339 PMC6887100

[43] Tanaka, T., Kohno, H., Suzuki, R., Yamada, Y., Sugie, S. and Mori, H. (2003) A novel inflammation-related mouse colon carcinogenesis model induced by azoxymethane and dextran sodium sulfate. Cancer Sci. 94, 965–973 10.1111/j.1349-7006.2003.tb01386.x 14611673 PMC11160237

[44] Chen, L., Wilson, J.E., Koenigsknecht, M.J., Chou, W.-C., Montgomery, S.A., Truax, A.D. et al. (2017) NLRP12 attenuates colon inflammation by maintaining colonic microbial diversity and promoting protective commensal bacterial growth. Nat. Immunol. 18, 541–551 10.1038/ni.3690 28288099 PMC5395345

[45] Man, S.M., Zhu, Q., Zhu, L., Liu, Z., Karki, R., Malik, A. et al. (2015) Critical role for the DNA sensor AIM2 in stem-cell proliferation and cancer. Cell 162, 45–58 10.1016/j.cell.2015.06.001 26095253 PMC4491002

[46] Dihlmann, S., Tao, S., Echterdiek, F., Herpel, E., Jansen, L., Chang-Claude, J. et al. (2014) Lack of absent in melanoma 2 (AIM2) expression in tumor cells is closely associated with poor survival in colorectal cancer patients. Int. J. Cancer 135, 2387–2396 10.1002/ijc.28891 24729378

[47] Wilson, J.E., Petrucelli, A.S., Chen, L., Koblansky, A.A., Truax, A.D., Oyama, Y. et al. (2015) Inflammasome-independent role of AIM2 in suppressing colon tumorigenesis by interfering with DNA-PK–dependent Akt activation. Nat. Med. 21, 906–913 10.1038/nm.3908 26107252 PMC4529369

[48] Chen, J., Wang, Z. and Yu, S. (2017) AIM2 regulates viability and apoptosis in human colorectal cancer cells via the PI3K/Akt pathway. OncoTargets Ther. 10, 811–817 10.2147/OTT.S125039 PMC5315344 28243117

[49] Shah, S., Qin, S., Luo, Y., Huang, Y., Jing, R., Shah, J.N. et al. (2021) AIM2 inhibits BRAF-mutant colorectal cancer growth in a caspase-1-dependent manner. Front. Cell Dev. Biol. 9, 588278 10.3389/fcell.2021.588278 33842454 PMC8027362

[50] Yang, Y., Zhang, M., Jin, C., Ding, Y., Yang, M., Wang, R. et al. (2019) Absent in melanoma 2 suppresses epithelial-mesenchymal transition via Akt and inflammasome pathways in human colorectal cancer cells. J. Cell. Biochem. 120, 17744–17756 10.1002/jcb.29040 31210372

[51] Karki, R., Man, S.M., Malireddi, R.K.S., Kesavardhana, S., Zhu, Q., Burton, A.R. et al. (2016) NLRC3 is an inhibitory sensor of PI3K-mTOR pathways in cancer. Nature. 540, 583–587 10.1038/nature20597 27951586 PMC5468516

[52] Karki, R., Malireddi, R.K.S., Zhu, Q. and Kanneganti, T.-D. (2017) NLRC3 regulates cellular proliferation and apoptosis to attenuate the development of colorectal cancer. Cell Cycle 16, 1243–1251 10.1080/15384101.2017.1317414 28598238 PMC5531621

[53] Yao, C., Hirata, T., Soontrapa, K., Ma, X., Takemori, H. and Narumiya, S. (2013) Prostaglandin E 2 promotes Th1 differentiation via synergistic amplification of IL-12 signalling by cAMP and PI3-kinase. Nat. Commun. 4, 1685 10.1038/ncomms2684 23575689 PMC3644078

[54] Mamantopoulos, M., Ronchi, F., Van Hauwermeiren, F., Vieira-Silva, S., Yilmaz, B., Martens, L. et al. (2017) Nlrp6- and ASC-dependent inflammasomes do not shape the commensal gut microbiota composition. Immunity 47, 339–348.e4 10.1016/j.immuni.2017.07.011 28801232

[55] Zaki, H., Boyd, K.L., Kastan, M.B., Lamkanfi, M. and Kanneganti, T.-D. (2010) The NLRP3 inflammasome protects against loss of epithelial integrity and mortality during experimental colitis. Immunity 32, 379–391 10.1016/j.immuni.2010.03.003 20303296 PMC2982187

[56] Kofoed, E.M. and Vance, R.E. (2012) NAIPs: building an innate immune barrier against bacterial pathogens. BioEssays 34, 589–598 10.1002/bies.201200013 22513803

